# Phytochemical Constituents of *Adansonia digitata* L. (Baobab) Fruit Pulp from Tekeze Valley, Tigrai, Ethiopia

**DOI:** 10.1155/2023/5591059

**Published:** 2023-10-25

**Authors:** Abebe Asmamaw Wasihun, Desta Berhe Sbhatu, Goitom Gebreyohannes Berhe, Kiros Hagos Abay, Gebreselema Gebreyohannes

**Affiliations:** ^1^Tigrai Biotechnology Center Pvt. Ltd. Co., Mekelle, Ethiopia; ^2^Department of Biological and Chemical Engineering, Mekelle Institute of Technology, Mekelle University, P.O. Box 1632, Mekelle, Ethiopia; ^3^Department of Chemistry, College of Natural and Computational Sciences, Mekelle University, P.O. Box 231, Mekelle, Ethiopia; ^4^Department of Materials Science and Engineering, Mekelle Institute of Technology, Mekelle University, P.O. Box 1632, Mekelle, Ethiopia

## Abstract

Baobab (*Adansonia digitata* L) is a large tree species growing in semiarid and arid lowlands of Ethiopia and other places. The plant is valued by natives for its contributions as a cash crop and livelihood tree. Previous studies using samples from different countries have documented their phytochemical profiles and nutritional and health benefits. This study explored the phytochemical constituents and biological activities of fruit pulp extracts of baobab collected from Tekeze Valley, Tigrai, Ethiopia. To this end, qualitative phytochemical screening tests, quantitative phytochemical analyses, and gas chromatography-mass spectrometry (GC-MS) analysis were carried out using aqueous extract. Analyses of antioxidant activities were also conducted with aqueous- and methanol-extracts using of 2,2-diphenyl-1-picrylhydrazyl (DPPH), nitric oxide (NO), and hydroxyl (OH) radical scavenging activity assays. The qualitative screening tests showed the presence of flavonoids, phenols, saponins, tannins, and terpenoids. Quantitative analyses of these phytochemicals at 25, 50, and 100 g/mL aqueous extract resulted in 0.0252 to 0.1000% yields. Yields of flavonoids, phenols, and saponins were higher at 50 g/mL extract, while that of tannins and terpenoids were higher at 100 g/mL. GC-MS analysis resulted in 15 predominant compounds including (1,2bis(trimethylsilyl)benzene (13.17%), 2-methyl-7-phenylindole (11.75%), 2-ethylacridine (10.11%), and benz[b]-1,4-oxazepine-4(5H)-thione,2,3-dihydro-2,8-dimethyl (10.11%). Aqueous and methanol extracts showed concentration-dependent antioxidant activities. In all the assays and concentrations, the antioxidant activities of both extracts were lower than that of the ascorbic acid standard. At equal extract concentrations (e.g., 100 and 250 *μ*g/mL), methanol extract had higher antioxidant activities than aqueous extract. The findings can encourage future initiatives towards large-scale research for compiling a complete phytochemical profile of the fruit pulp of the Ethiopian baobab.

## 1. Introduction

Baobab (*Adansonia digitata* L) (Malvaceae) is a large, conspicuous tree, native to semiarid regions of Africa, Asia (in China and Malaysia), Australia, and the Caribbean (especially Jamaica). Locally called *Dima* (in Tigrinya), the plant is very common in deserts and hot lowlands in Ethiopia. The tree is highly valued by rural communities of arid and hot lowlands of sub-Saharan Africa for its fruits and edible leaves. It is an important cash crop, a source of healthy human food and nutrition with medicinally and pharmaceutically important constituents, and livestock fodder [1–6]. Unfortunately, it is a neglected, locally and globally endangered tree species with no strategies for its conservation, natural and artificial regeneration, and sustainable use [3].

Previous studies in baobab-growing countries have shown that the fruit is the source of nutritionally and medicinally important compounds [7–9]. It is, thus, called the chemist tree because of its health benefits [2, 10, 11]. Baobab fruit pulp has very high vitamin C content (ca. ten times that of orange) [5]. Its seeds have substantial quantities of crude protein, digestible carbohydrates, and oil and high levels of lysine, thiamine, calcium, and iron [5, 6]. Likewise, baobab leaves are superior in nutritional quality to fruit pulp and contain significant vitamin A levels. All parts of the plant have many phytochemical constituents with multiple biological properties including antioxidant and anti-inflammatory activities [5, 12]. Seed, pulp, and seed oil of baobab are also good sources of macro- and micronutrients such as potassium, magnesium, calcium, and phosphorus as well as many health-promoting substances [11, 13]. Hence, the fruit pulp (powder) can be consumed fresh or as a traditionally and industrially processed product. It also has a huge potential for large-scale industrial utilization via producing functional foods and beverages [14, 15].

However, despite the plant grows in the vast area of hot lowlands in Ethiopia, in general, and in Tigrai (northern Ethiopia), in particular, no scientific study was carried out before to explore its chemistry, conservation, and natural and artificial regeneration. No efforts were also made towards its large-scale industrial application. Therefore, the present study aims to describe the qualitative and quantitative phytochemical constituents, identify the major compounds using GC-MS and other methods, and examine the antioxidant properties of the baobab fruit pulp extract. The study's findings will contribute to initiating future research and development programs towards the sustainable use of the plant.

## 2. Materials and Methods

### 2.1. Collection Site of Baobab Fruits

Baobab fruits used in the study were collected from Tekeze Valley (11°40′ and 15°12′N and 36°30′ and 39°50′E), Western Zone of Tigrai Region, Ethiopia. The site of fruit collection is located 96 km southwest of Shire-Endaselassie city, stretching between the Asgede-Tsimbla district to the east and the Wolqait district to the west [16].

### 2.2. Collection and Preparation of Fruits

Fruits were collected during the fruit ripening season of December 2019. Collection of biological materials by Ethiopian researchers for research and development purposes is granted by Article 15, Clause 1 of the Access to Genetic Resources and Community Knowledge, and Community Rights Proclamation of Ethiopia (No. 482/2006). Healthy, ripened, and brownish fruits were collected from five mature trees of riverine forest. The total density in the riverine forest of the area is 5(±0.8) stems per hectare according to one recent study [17]. The fruits collected from each tree were counted and recorded. The fruits were packed in plastic bags and shipped to the tissue culture laboratory of Tigrai Biotechnology Center Pvt. Ltd. Co, Mekelle, Ethiopia, and stored in dry storage boxes until use. Then, healthy, dry fruits with no discoloration were carefully selected and washed using warm tap water (50°C) and sodium hypochlorite to remove any soil, debris, and microbial contaminants. The washed fruits were immediately put within a laminar air flow (LAF) cabinet and were allowed to fully dry before being processed [9].

### 2.3. Separation of Fruit Pulp

The hard woody shells of the fruits were removed using a sterilized stainless steel knife inside a LAF cabinet. This procedure yielded the white pulp (powder) of the baobab fruit holding several seeds and fibers. The pulp was, then, separated from the seeds and fibers by smashing with a pestle and collected into a sterilized and clean mica bowl. The powdery pulp was sieved using 0.9 mm pore mesh. The pure and fine pulp was transferred into a sterilized clean jar, tightly closed, and kept in a dark and cool place until used for further analyses [9].

### 2.4. Extraction of *A. digitata* Fruit Pulp

Extraction of fruit pulp was carried out at the geochemical laboratory of the College of Natural and Computational Sciences, Mekelle University, Mekelle, Ethiopia. A 10.0 g sample of dried baobab fruit pulp was macerated in 100.0 mL distilled water with continuous stirring for 24 h. Then, the mixture was filtered and concentrated using a rotary evaporator at 40°C under reduced pressure yielding aqueous extract. Likewise, a 20 g sample of the pulp was macerated in 150.0 mL of 80% methanol through continuous steering for 72 h. Then, the mixture was filtered, concentrated, and dried using a rotary-evaporator at 40°C yielding the methanol extract. The concentrated aqueous and methanol extracts were kept in a refrigerator at 4°C until further analyses [18].

### 2.5. Qualitative Phytochemical Screening

#### 2.5.1. Alkaloids

Qualitative analysis of alkaloids was carried out using Mayer's test as described in the work of Ansari [19]. A 5.0 mL baobab pulp aqueous extract was evaporated in a test tube leaving a residue. A 1.0 mL of 5% (v/v) HCl was added to the residue in the test tube, shaken well, and filtered. Then, 10 drops of Mayer's reagent were added to the filtrate. The formation of a yellow precipitate signifies a positive test for alkaloids.

#### 2.5.2. Flavonoids

Flavonoids were screened using the Shinoda test as described by Kokate [20]. A 10.0 mL/g aqueous extract of baobab fruit pulp was added to a test tube, and 5.0 mL 95% ethanol and a few drops of conc. HCl were added to it. Then, 0.50 g Mg chips were added to the solution. Pink coloration indicates a positive test for flavonoids.

#### 2.5.3. Glycosides

Identification of glycosides was carried out using the Keller–Killiani test. A 2.0 mL aqueous extract of baobab fruit pulp was added to a test tube. Then, 1.0 mL glacial acetic acid, 1 drop of 5% FeCl_3_, and 1.0 mL conc. H_2_SO_4_ were added. The appearance of reddish-brown color at the junction of two liquid layers and the turning of the upper layer into bluish-green indicate the presence of glycosides [19].

#### 2.5.4. Phenols

Screening for phenols was carried out using the ferric chloride test [21]. A 2.0 mL aqueous extract of baobab fruit pulp was added to a test tube. Then, it was diluted to 5.0 mL by adding distilled water, and a few drops of neutral 5% FeCl_3_ solution were added to it. A dark green color indicates a positive test.

#### 2.5.5. Saponins

The screening for saponins was carried out using the foam test [19]. A 5.0 mL baobab pulp aqueous extract was mixed with 5.0 mL distilled water and shaken vigorously for 10 min. The development of stable foam indicates a positive test for saponins.

#### 2.5.6. Steroids

Qualitative analysis of steroids was carried out using the Salkowski test [22]. A 2.0 mL aqueous fruit pulp extract of baobab was added to a test tube. Then, 2.0 mL chloroform and 2.0 mL conc. H_2_SO_4_ were added to it, and the solution was shaken well. The turning of the chloroform layer into red and the acid layer into greenish-yellow fluorescence signifies a positive test for steroids.

#### 2.5.7. Tannins

The screening for the presence of tannins was carried out using the lead acetate test [21]. A 5.0 mL aqueous extract of baobab was added to a test tube, and 2.0 mL lead acetate solution was added to it. The development of a white precipitate signifies a positive test for tannins.

#### 2.5.8. Terpenoids

The procedure for detecting terpenoids was carried out using the Salkowski reaction with some modifications [23]. A 0.15 g baobab fruit pulp aqueous extract was mixed with 2.0 mL chloroform followed by careful addition of 4.0 mL conc. H_2_SO_4_. The mixture was allowed to form a layer, and a reddish-brown coloration in the interface indicates the presence of terpenoids.

### 2.6. Quantitative Phytochemical Analyses

#### 2.6.1. Total Flavonoid Content

The total flavonoid content (TFC) of the fruit pulp of *A. digitata* was determined according to the aluminum chloride method using catechin as a standard [24]. A 1.0 mL aqueous extract was poured into a volumetric flask and mixed with 4.0 mL distilled water and was allowed to stand for 5 min. Then, 0.30 mL 5% NaNO_2_ and 0.30 mL 10% AlCl_3_ were added and the mixture and was left for 6 min at room temperature. A 2.0 mL 1.0 M NaOH was added to the reaction mixture, and some distilled water was immediately added until the mixture reached the 10.0 mL mark. The absorbance of the reaction mixture was measured using a UV-vis spectrophotometer (Lambda, CE1021, Australia) at 510 nm against a blank. A calibration curve was generated using 10–100 *μ*g/mL of the standard catechin solution (*R*^2^ = 0.991). The TFC values were calculated based on the curve, and the contents were expressed as mg of catechin equivalent per gram of the dried extract (mg CE/g dried extract).

#### 2.6.2. Total Phenolic Content

The total phenolic content (TPC) of the fruit pulp aqueous extract was determined using the Folin–Ciocalteu reagent (FCR) [24]. A 1.0 mL aqueous extract of different concentrations was mixed with 0.40 mL FCR (diluted 1 : 10 v/v) in volumetric flasks and was allowed to stand for 5 min. Then, 4.0 mL of 7% Na_2_CO_3_ solution was added. Upon reaching the 10.0 mL mark by adding distilled water, the mixture was allowed to stand for 90 min at room temperature. The absorbance of each of the samples was measured against a blank at 750 nm using a UV-vis spectrophotometer. A calibration curve was generated using 20–200 *μ*g/mL of the standard gallic acid solution (*R*^2^ = 0.998). The TPC values were calculated based on the curve of gallic acid solution. The contents were expressed as mg of gallic acid equivalent per g of the dried extract (mg GAE/g dried extract).

#### 2.6.3. Total Saponin Content

The total saponin content (TSC) was estimated according to the procedure established in the works of Sim [25]. A 1.0 mL sample of aqueous extract was put into a test tube and was mixed with 2.0 mL 8% vanillin, dissolved in ethanol, and agitated until it forms a homogeneous solution. Then, 2.0 mL 72% H_2_SO_4_ was added to the solution, mixed well, heated in a water bath at 60°C for 10 min, and allowed to cool. The absorbance of the solution was measured at 544 nm against a blank using a UV-vis spectrophotometer. A calibration curve was generated using 10–100 *μ*g/mL of Diosgenin standard solution (*R*^2^ = 0.992). The TSC values were calculated based on this calibration curve. The contents were expressed as mg of Diosgenin equivalents per g of the dried extract (mg DE/g dried extract).

#### 2.6.4. Total Tannin Content

The total tannin content (TTC) of the *A. digitata* fruit pulp extract was determined by using tannic acid as a reference compound according to the method described by Saeed et al. [24]. A 1.0 mL sample was put into a test tube and mixed with 5.0 mL vanillin hydrochloride reagent (comprising equal volumes of 8% HCl in methanol and 4% vanillin in methanol). The mixture was allowed to stand for 20 min to complete the reaction, and its absorbance was measured at 500 nm using a UV-vis spectrophotometer. A calibration curve was generated using 20–200 *μ*g/mL of tannic acid standard solution (*R*^2^ = 0.991). The TTC values were calculated based on this calibration curve. The contents were expressed as mg of tannic acid equivalents per g of the dried extract (mg TAE/g dried extract).

#### 2.6.5. Total Terpenoid Content

The total terpenoid content was determined according to the method described by Ghorai et al. [26]. A 1.0 mL sample of the aqueous fruit pulp extract was prepared in a test tube, and 3.0 mL chloroform was added to it. The mixture was thoroughly vortexed, left for 3 min, and 200 *μ*L conc. H_2_SO_4_ was added to it. The mixture was incubated at room temperature for 1.5–2 h in dark to form a reddish-brown precipitate. Then, all the supernatant of the reaction mixture was carefully and gently decanted without disturbing the precipitate. At the end, 3.0 mL 95% (v/v) methanol was added to the precipitate and vortexed thoroughly until the precipitate was dissolved completely. The absorbance was measured at 538 nm using a UV/vis spectrophotometer. A calibration curve was generated using 10–100 *μ*g/mL of linalool standard solution (*R*^2^ = 0.994). The values of total terpenoid content were calculated based on this calibration curve. The contents were expressed as mg of linalool equivalents per g of the dried extract (mg LE/g dried extract).

### 2.7. GC-MS Analysis of Aqueous Extract of *A. digitata* Fruit Pulp Powder

Chemical constituents of the aqueous extract of the baobab fruit pulp were analyzed and identified using a gas chromatography-mass spectrometer (GC-MS) according to the method developed by Salim [27]. A 2.0 *μ*L sample of the aqueous pulp extract was dissolved in HPLC-grade aqueous solution and subjected to GC. An Agilent 7820AGC system was used for the GC. DB-5 column fused with silica (50 m length × 0.25 mm internal diameter) was used for separation. The column temperature was set to 100°C for 20 min and increased to 270°C for 3 min. Helium was used as the carrier gas with a split ratio of 5 : 4. A 1.0 *μ*L sample was evaporated in a splitless injector at 300°C in 22 min run time. The molecular weight, molecular formula, and structure of the compounds were ascertained by interpretation of the mass spectrum of GC-MS using the database of the NIST library and relevant literature.

### 2.8. Antioxidant Properties of Extracts of *A. digitata* Fruit Pulp Powder

#### 2.8.1. DPPH Radical Scavenging Activity Assay

The free radical scavenging activities of aqueous and methanol extracts were measured *in vitro* by the 2,2′-diphenyl-1-picrylhydrazyl (DPPH) assay according to a standard method [28]. A stock solution was prepared by dissolving 24.0 mg DPPH in 100.0 mL ethanol and was stored at 20°C. A working solution was prepared by diluting DPPH stock solution in ethanol, and a 3.0 mL aliquot of the working solution was mixed with 1.0 mL aqueous pulp extract at 100, 250, 500, 750, and 1,000 *μ*g/mL and methanol pulp extract at 50, 100, 150, 200, and 250 *μ*g/mL. Reaction mixtures were shaken well and incubated in the dark for 15 min at room temperature. The absorbance of each mixture was taken at 517 nm using a UV-vis spectrophotometer. A control sample was prepared without the extract, and scavenging activity was estimated based on the percentage of the DPPH radical scavenged as % Inhibition *=* [(Control OD − Sample OD)/(Control OD] × 100, where OD refers to the optical density.

#### 2.8.2. Nitric Oxide Scavenging Activity

The nitric oxide radical scavenging (NOS) activities of the extracts were determined according to the method described by Erwa and coworkers[29]. A 1.0 mL of 10 mM sodium nitroprusside in phosphate-buffered saline was mixed with 1.0 mL pulp extract of different concentrations (i.e., 100, 250, 500, 750, and 1,000 *μ*g/mL for the aqueous extract and 50, 100, 150, 200, and 250 *μ*g/mL for the methanol extract) and incubated at 25°C for 180 min. Then, 1.0 mL Griess reagent (a mixture of an equal volume of 1% sulphanilamide, 0.1% naphthyl-ethylenediamine dichloride, and 3% H_2_PO_4_) was added to the incubated solution, and the absorbance was read at 546 nm using a UV-vis spectrophotometer. Ascorbic acid was used as positive control and treated in the same way as the Griess reagent. The percentage inhibition was calculated as Scavenging Effect (%) = [(Control OD − Sample OD)/(Control OD] × 100, where OD refers to the optical density.

#### 2.8.3. Hydroxyl Radical Scavenging Activity

Determinations of hydroxyl radical scavenging (HRS) activities of the extracts were carried out as per the method described in Saeed et al. [24]. Reaction mixtures comprising 0.8 mL phosphate buffer solution (50 mmol/L, pH 7.4), 0.20 mL extract of the different concentrations (i.e., 100, 250, 500, 750, and 1,000 *μ*g/mL aqueous pulp extract and 50, 100, 150, 200, and 250 *μ*g/mL methanol pulp extract), 0.20 mL EDTA (1.04 mmol/L), 0.20 mL FeCl_3_ (1.0 mmol/L), and 0.20 mL of 2-deoxyribose (60 mmol/L) were prepared in test tubes. The mixtures were kept in a water bath at 37°C. The reaction of each mixture was initiated by adding 0.20 mL ascorbic acid (2.0 mmol/L) and 0.20 mL H_2_O_2_ (10.0 mmol/L) and was left for 1 h. Then, 2.0 mL cold thiobarbituric acid (10 g/L) and 2.0 mL HCl (25%) were sequentially added to each of the reaction mixtures. The reaction mixtures were heated at 100°C for 15 min and were cooled in a cold water bath. The absorbance of each solution was measured at 532 nm using a UV-vis spectrophotometer. The HRS capacity was evaluated with the inhibition percentage of 2-deoxyribose oxidation on hydroxyl radicals. The scavenging percentage was calculated as Scavenging Effect (%) = [(Control OD − Sample OD)/(Control OD)] × 100, where OD refers to the optical density.

### 2.9. Data Analyses

All the tests, experiments, and measurements were carried out in triplicate. The data were analyzed by inferential statistical methods using SPSS Version 20 software. Inferential (sample) data were processed using the analysis of variance (ANOVA) at an *a priori* set *p* value of ≤0.05. Post-hoc comparisons of the mean (±SD) values were carried out using the least significance difference (LSD). Qualitative data collected by visual observations were used to strengthen the results of the quantitative data analyses.

## 3. Results and Discussion

### 3.1. Phytochemical Study of *A. digitata* Fruit Pulp Powder

#### 3.1.1. Qualitative Screening

The qualitative phytochemical screening assay of baobab fruit pulp powder was conducted using the aqueous extract. The assay revealed that five of the eight sought phytochemical groups, namely, flavonoids, phenols, saponins, tannins, and terpenoids were detected in the aqueous extract (Table 1). Previous studies, that employed different methods and extraction solvents, have reported the presence of all or some of these chemical groups [14, 30–33]. The presence of flavonoids and saponins, which promote the antioxidant properties of wild fruits, can extend the shelf lives of derived foods, beverages, and cosmetics [14, 34, 35]. Alkaloids, flavonoids, phenols, saponins, tannins, and terpenoids are important phytochemical constituents with high antioxidant properties and many other therapeutic uses. They are also known for their antimicrobial properties and cholesterol-lowering effects [35–41]. Secondary metabolites, with these and other health benefits by reducing the risks of several chronic diseases, are common in fresh fruits and other plant products. These findings encourage comprehensive research to investigate the Ethiopian baobab fruit pulp for medicinal and nutritional phytochemicals.

#### 3.1.2. Quantitative Analysis

Quantitative analyses of the phytochemicals were carried out with 25, 50, and 100 g/mL fruit pulp extract concentrations. The mean (±SD) concentrations of the phytochemicals increased at statistically significant levels with increasing the concentrations of the extract (Table 2; *p* ≤ 0.05). But when the yields are compared, the yields of flavonoids (0.0732%), phenols (0.0652%), and saponins (0.0386%) are higher with 50 g/mL extract, while those of tannins (0.1000%) and terpenoids (0.0636%) are higher with 100 g/mL extract. The lowest yields were observed with 25 g/mL extract in all the phytochemical groups except in flavonoids (Table 2). Thus, higher yields of the phytochemical were observed with 50 and 100 g/mL extract.

A study on aqueous fruit extract of Nigerian baobab showed 16.14 mg/g flavonoids, 100.00 mg/g saponins, 351.0 mg/g tannins, and 70.00 mg/g alkaloids [30]. Another study on the hexane fruit extract of Senegalese baobab yielded 5.66 ± 0.18 *μ*g/mg total flavonoids, 103.09 ± 0.63 *μ*g/mg total tannins, and 27.21 ± 0.26 mg/g total polyphenols [33]. A chemical analyses study with aqueous extracts of Saudi Arabian baobab fruit reported 42.70 ± 0.43 mg/g flavonoid and 48.08 ± 1.08 mg/g phenolic contents [42]. Also, a study on fruits extracts collected from various parts of Sudan revealed phenolic contents of 15.50 to 99.66 mg GAE/g and flavonoid contents of 1.03 to 21.53 mg CE/g [43]. A study on the fruit extract of Malawian baobab found a total phenolic content of 1,870 ± 1.61 mg/100 g fresh weight [35]. Such high total phenolic contents were also reported for fruit pulp extracts from Madagascar (1,090 mg GAE/100 g) [44] and Burkina-Faso (3,520 to 4,060 mg GAE/100 g) [45]. Therefore, with 100 g/mL fruit pulp extract, the flavonoid content obtained in the present study was higher than those reported for the Nigerian, Saudi Arabian, Senegalese, and Sudanese baobab fruit pulps. Moreover, the phenolic content was higher than those reported for fruit pulps from Burkina Faso, Malawi, Madagascar, Saudi Arabia, and Senegal. However, the saponin and tannin contents were lower than those reported for the Nigerian and Senegalese fruit pulps.

### 3.2. GC-MS Analysis *A. Digitata* Aqueous Fruit Pulp Extract

**Figure 1:**
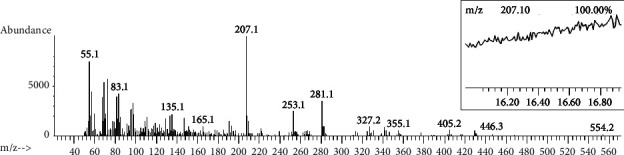
Gas chromatography-mass spectrometry chromatogram of the bioactive compounds in the aqueous extract of *Adansonia digitata* L. fruit pulp.

Baobab is known to be the source of several secondary metabolites with multiple nutritional, biological, and pharmaceutical activities and properties [11, 35, 40, 41, 46, 47]. A study on a Malian baobab fruit pulp using high performance liquid chromatography coupled with a photodiode array/UV and electrospray ionization-mass spectrometer and mass spectrometer (HPLC-PDA/UV-ESI-MS/MS) analyses detected citric acid and 14 phenolic compounds [11]. Another HPLC-based study on Malawian fruit also detected high quantities of vitamin C, multiple organic acids, and phenolic compounds [35]. One study on fruit pulp of Nigerian baobab using GC-MS analysis [40] and another on Sudanese baobab using the LC-MS/MS analysis [46] detected 36 and 52 bioactive compounds, respectively. A recent study on the fruit extract using ultrahigh performance liquid chromatography coupled to high-resolution tandem mass spectrometry (UHPLC-HRMS/MS), headspace solid-phase microextraction/gas chromatography coupled to mass spectrometry (HS-SPME/GC-MS), and GC-MS postsilylation detected 77 compounds [41]. In the present study, the GC-MS analysis of the aqueous extract of the Ethiopian baobab fruit pulp yielded a chromatogram with greater than 200 picks. Fifteen predominant chemical compounds with an area of 5.68 to 13.17% were identified based on the reference compound at pick *m*/*z* 207.10 (100.00%) (Figure 1, Table 3, and Supplementary file, Figure S1). The choices of extraction solvent, retention time, area (%), GC-MS column, reference compound, and NIST data interpretation system determine the qualitative and quantitative results of the GC-MS and other spectrometer methods [45, 46]. The literature study showed that each of the 14 chemical compounds has multiple biological and pharmaceutical activities and/or properties [48–62].

### 3.3. Antioxidant Properties of *A. digitata* Fruit Pulp Extracts

The antioxidant activities of aqueous and methanol extracts of baobab fruit pulp, analyzed as functions of DPPH radical scavenging, HRS, and NOS showed concentration-dependent increments (*p* ≤ 0.05) (Table 4). Concentration-dependent increments of antioxidant activities were also reported by many other researchers [10, 14, 63, 64]. Maximum antioxidant activities of aqueous extract were observed in HRS (62.00 ± 1.41%) followed by DPPH (53.60 ± 0.81%) and NOS (51.60 ± 1.08%) at 1,000 *μ*g/mL. But with the methanol extract, maximum activities were observed in DPPH (72.3 ± 1.08%) followed by HRS (70.6 ± 1.08%) and NOS (68.6 ± 1.47%) at 250 *μ*g/mL. The antioxidant activities of the aqueous and methanol extracts at all concentrations were weaker than that of the ascorbic acid standard except for the methanol extract at 50 *μ*g/mL. The differences between the antioxidant activities of both extracts and the ascorbic acid standard were narrowed down at higher concentrations. Such a trend is a commonly reported observation and is linked to the performance and amount of the antioxidants [63, 64]. The reducing ability of extracts generally depends on the presence of antioxidants (reductones) that exert antioxidant activities by breaking free radical chains by donating hydrogen atoms [65]. But as the reactions progress, the antioxidants can be depleted and become limiting factors for the antioxidant activities. Moreover, the activities of such extracts decline after they passed the inhibitory concentration (IC_50_) levels of the scavenging processes because the number of free radicals exceeds the number of antioxidants [24].

The present study showed stronger antioxidant activities with the methanol extract. At 100 *μ*g/mL, the antioxidant activities of the methanol extract were 2.77 times stronger for DPPH radical scavenging, 2.68 times stronger for HR scavenging, and 4.08 times stronger for NO scavenging than the aqueous extract. The antioxidant activities at 250 *μ*g/mL were also 3.14 times stronger for DPPH radical scavenging, 2.08 times stronger for HRS, and 5.81 times stronger for NOS with the methanol extract compared to the aqueous extract. Even with the ascorbic acid standard, the antioxidant activities of the methanol extract at 100 *μ*g/mL and 250 *μ*g/mL were 1.14 and 1.43 times stronger, respectively. Even if the concentration of the aqueous extract was four times (i.e., 1,000 *μ*g/mL) than that of the methanol extract (i.e., 250 *μ*g/mL), its antioxidant activities were weaker by about 10–20%. In line with this finding, many studies have shown that types of solvents and further fractionation of the extracts using various solvents affect the antioxidant activities of the fruit pulp extracts [40, 66–68].

Several studies have also shown that the methods of the assay (analysis) [11, 33, 64, 67, 69] and the geographical location or ecology of the baobab fruit [11, 68] affect the antioxidant activities of the pulp extracts. In one study, assays of scavenging capacities were carried out using DPPH, ABTS (2,2′-azinobis-(3-ethylbenzothiazoline-6-sulfonic acid) diammonium salt)), and nitrite methods with n-hexane, ethyl acetate, chloroform, n-butanol, and residual aqueous fractions of the aqueous fruit extract. Whereas DPPH yielded the best performance with *n*-hexane fraction, ABTS and nitrite yielded best performances with n-butanol and ethyl acetate, respectively [67]. Likewise, a study by Braca et al. [11] using n-butanol fruit pulp extract reported better performance with the ABTS assay compared to DPPH and FRAP (ferric reducing antioxidant power) assays. The present study revealed that HRS was the best method of assay in showing stronger antioxidant activities with the aqueous fruit extract. With the methanol extract, the DPPH and HRS methods were comparable and better than the NOS.

## 4. Conclusion

The present study has provided us with important data about the phytochemical constituents of the Ethiopian baobab fruit pulp collected from Tekeze Valley. The aqueous extract of the fruit pulp was the source of flavonoids, phenols, saponins, tannins, and terpenoids. Stronger antioxidant activities were observed with the methanol extract than with the aqueous extract. GC-MS analysis generated 15 predominant compounds where 14 of them have multiple biological and pharmaceutical activities and properties. The differences in antioxidant activities due to differences in extraction solvents and methods of free radical scavenging assays would call for a further comprehensive study to develop a complete phytochemical profile of the Ethiopian baobab fruit pulp.

## Figures and Tables

**Table 1:** Phytochemical screening of crude aqueous extract of *A. digitata* fruit pulp compared with previous studies.

No.	Phytochemicals	This study	[14]	[30]	[31]	[32]	[33]
1	Alkaloids	−(WE)	+(WE)	+(WE)	−(WE)	+(AE)	+(ME)
2	Flavonoids	+(WE)	+(WE)	+(WE)	−(WE)	+(ME)	+(ME)
3	Glycosides	−(WE)	+(WE)	+(WE)	+(WE)	+(ME)	NA
4	Phenols	+(WE)	NA	+(WE)	NA	NA	NA
5	Saponins	+(WE)	+(WE)	+(WE)	+(WE)	+(ME)	+(ME)
6	Steroids	−(WE)	NA	+(WE)	+(WE)	NA	+(ME)
7	Tannins	+(WE)	+(WE)	+(WE)	+(WE)	+(AE)	+(ME)
8	Terpenoids	+(WE)	+(WE)	+(WE)	+(WE)	+(HE)	NA

−: absent, +: present; NA: not analyzed; AE: acetone extract, HE: hexane extract, ME: methanol extract, WE: water extract.

**Table 2:** Quantitative analyses and yield of phytochemicals of *A. digitata* fruit pulp aqueous extract.

No	Phytochemicals	Yield in mg/g (mean ± SD values and percentages)					
		25 g/mL extract		50 g/mL extract		100 g/mL extract	
		Mean ± SD	%	Mean ± SD	%	Mean ± SD	%
1	Flavonoids	15.60 ± 0.08^c^	0.0624	36.60 ± 1.08^b^	0.0732	55.30 ± 1.04^a^	0.0553
2	Phenols	11.00 ± 0.70^c^	0.0440	32.60 ± 1.08^b^	0.0652	50.30 ± 1.08^a^	0.0503
3	Saponins	6.30 ± 1.08^c^	0.0252	19.30 ± 1.47^b^	0.0386	31.60 ± 1.08^a^	0.0316
4	Tannins	16.30 ± 1.08^c^	0.0652	40.30 ± 1.08^b^	0.0806	100.00 ± 1.4^a^	0.1000
5	Terpenoids	11.00 ± 0.70^c^	0.0440	27.60 ± 1.08^b^	0.0552	63.60 ± 1.0^a^	0.0636

Means (± SD) in the same row with different letters are statistically significantly different at *p* ≤ 0.05.

**Table 3:** Predominant chemical compounds in the aqueous extract of *Adansonia digitata* L. fruit pulp identified by gas chromatography-mass spectrometry (GC-MS).

SN	MF	Compound	Structure	MW	RT (min)	Area (%)	*m*/*z*+	Pharmaceutical or biological activities/properties	Ref
1	C_15_H_13_N	2-Ethylacridine	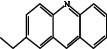	207.27^*∗*^	16.327	10.11	62, 96, 166	(i) Antitumor (ii) Antioxidant	[48, 49]

2	C_11_H_13_NOS	Benz[b]-1,4-oxazepine-4(5H)-thione,2,3-dihydro-2,8-dimethyl	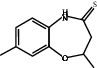	207.29	16.327	10.11	41, 85, 134, 174	(i) Anticonvulsants (ii) Muscle-relaxant (iii) Daytime sedative (iv) Tranquilizers (v) Anesthetics	[50, 51]

3	C_15_H_13_N	Benzo[h]quinoline,2,4-dimethyl	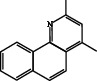	207.27^*∗*^	16.913	8.03	76, 127, 165	(i) Anticancer (ii) Antibacteria1 (iii) Antifunga1 (iv) Antimalarial	[49]

4	C_6_H_18_O_3_Si_3_	Cyclotrisiloxane, hexamethyl-	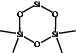	222.4618	16.431	9.54	96, 133, 177	(i) Antibacterial (ii) Antimicrobial (iii) Antitumor	[52, 53]

5	C_7_H_6_N_2_S	1,2-Benzisothiazol-3-amine	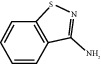	150.2	16.431	7.00	41, 74, 177	(i) Antimicrobial (ii) Antitumor (iii) Promotes human growth and development (iv) Treats skin cancer, atherosclerosis, and migraines (v) Reduces risk of heart disease	[54]

6	C_15_H_13_N	5-Methyl-2-phenylindolizine	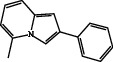	207.27^*∗*^	16.525	8.92	77, 130, 178	(i) Antibacterial (ii) Antitumor (iii) Treats dysentery and diarrhea	[55, 56]

7	C_12_H_18_O_2_Si	Propiophenone2-(trimethylsiloxy)	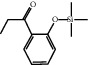	222.356	16.705	9.47	45, 75, 151, 177	(i) Antifungal (ii) Anti-leshmanial (iii) Insecticidal	[49, 57]

8	C_14_H_22_O_2_	2,4-Cyclohexadien-1-one, 3,5- bis(1,1-dimethylethyl)-4-hydroxy	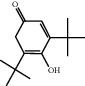	222.32	16.705	9.47	57, 123, 179	Not reported	

9	C_15_H_13_N	1H-Indole, 2-methyl-3-phenyl	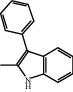	207.27^*∗*^	16.705	9.47	30, 77, 130, 178	(i) Anti-inflammatory (ii) Analgesic (iii) Immune-modulatory (iv) Antioxidant	[58, 59]

10	C_16_H_48_O_7_Si_8_	Octasiloxane, 1,1,3,3,5,5,7,7,9,9,11,11,13,13,15, 15-hexadecamethyl	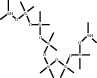	577.2	16.866	9.02	73, 147, 177	(i) Antioxidant (ii) Antibacterial	[48]

11	C_15_H_13_N	1H-Indole, 1-methyl-2-phenyl	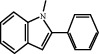	207.27^*∗*^	16.781	5.68	27, 63, 165	(i) Anti-inflammatory (ii) Analgesic	[49, 59]

12	C_10_H_30_O_3_Si_4_	Methyltris(trimethylsiloxy)silane	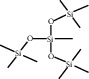	310.68	16.979	7.30	73, 133, 295	(i) Antioxidant (ii) Antibacterial (iii) Anti-inflammatory	[52]

13	C_12_H_22_Si_2_	1,2Bis(trimethylsilyl)benzene	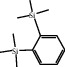	222.47	17.111	13.17	43, 73, 119	(i) Antibacterial (ii) Antifungal (iii) Anti-inflammatory	[58, 60]

14	C_15_H_13_N	2-Methyl-7-phenylindole	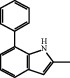	207.27^*∗*^	17.263	11.75	30, 102, 165	(i) Colorimetric lipid peroxidation assay (ii) Anti-inflammatory (iii) Antibacterial	[58, 60]

15	C_10_H_13_NO_2_	Propanamide, *N*-(4-methoxyphenyl)	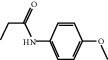	179.2	17.329	7.00	57, 120, 123, 164	(i) Formyl peptide receptor-2 agonists (ii) Antibacterial	[61, 62]

MF: molecular formula; MW: molecular weight; RT: retention time; *m*/*z*+: major fragments; Ref.: references; ^*∗*^: reference compound.

**Table 4:** Antioxidant activities of the *A. digitata* fruit pulp extract.

Extracts	Concentration (*μ*g/mL)	Mena (±SD) antioxidant activities of fruit pulp extract (%)			
		DPPH	HRS	NOS	AAS
Aqueous	100	12.00 ± 0.70^e^	13.00 ± 0.70^e^	8.00 ± 0.70^e^	44.30 ± 1.47^e^
	250	23.00 ± 0.70^d^	34.00 ± 1.41^d^	11.80 ± 0.54^d^	61.30 ± 1.08^d^
	500	35.00 ± 0.70^c^	41.00 ± 0.70^c^	16.00 ± 0.70^c^	69.30 ± 1.08^c^
	750	44.60 ± 1.08^b^	52.30 ± 1.08^b^	30.00 ± 0.70^b^	72.30 ± 1.47^b^
	1,000	53.60 ± 0.81^a^	62.00 ± 1.41^a^	51.60 ± 1.08^a^	77.60 ± 1.08^a^

Methanol	50	22.10 ± 0.73^e^	19.50 ± 0.35^e^	18.00 ± 0.70^e^	21.00 ± 0.70^e^
	100	33.20 ± 0.76^d^	34.90 ± 0.60^d^	32.60 ± 0.81^d^	50.60 ± 1.08^d^
	150	50.00 ± 0.70^c^	48.00 ± 0.70^c^	42.16 ± 0.73^c^	65.10 ± 0.54^c^
	200	62.10 ± 0.73^b^	64.00 ± 0.70^b^	56.60 ± 1.08^b^	76.90 ± 0.63^b^
	250	72.30 ± 1.08^a^	70.60 ± 1.08^a^	68.60 ± 1.47^a^	87.60 ± 1.08^a^

DPPH: 2,2′-diphenyl-1-picrylhydrazyl; HRS: hydroxyl radical scavenging; NOS: nitric oxide scavenging; AAS: ascorbic acid standard. Means (±SD) in the same column with different letters are significantly different at *p* ≤ 0.05.

## Data Availability

The datasets used and/or analyzed during the current study are available from the first author upon reasonable request.

## Supplementary Materials

Supplementary Materials This supplementary material (Figure S1) carries the entire GC-MS output from which the compounds indicated in Table 3 are identified.
