# Supplementary material for: Phytochemical Constituents of Adansonia digitata L. (Baobab) Fruit Pulp from Tekeze Valley, Tigrai, Ethiopia
Source: Int J Anal Chem. 2023 Oct 25;2023:5591059. doi: 10.1155/2023/5591059 (PMC10620028; doi:10.1155/2023/5591059)
Supplement: Supplementary Materials — This supplementary material (Figure S1) carries the entire GC-MS output from which the compounds indicated in Table 3 are identified. [file 5591059.f1.pdf]

Library Search Report

Data Path : D:\Data\anjac\2015\ANJAC\noorul islam univ\
Data File : S4.D
Acq On : 10 Feb 2020 12:29
Operator :
Sample : S4
Misc :
ALS Vial : 6 Sample Multiplier: 1

Search Libraries: D:\MassHunter\Library\NIST11.L Minimum Quality: 0

Unknown Spectrum: Apex
Integration Events: ChemStation Integrator - autoint1.e

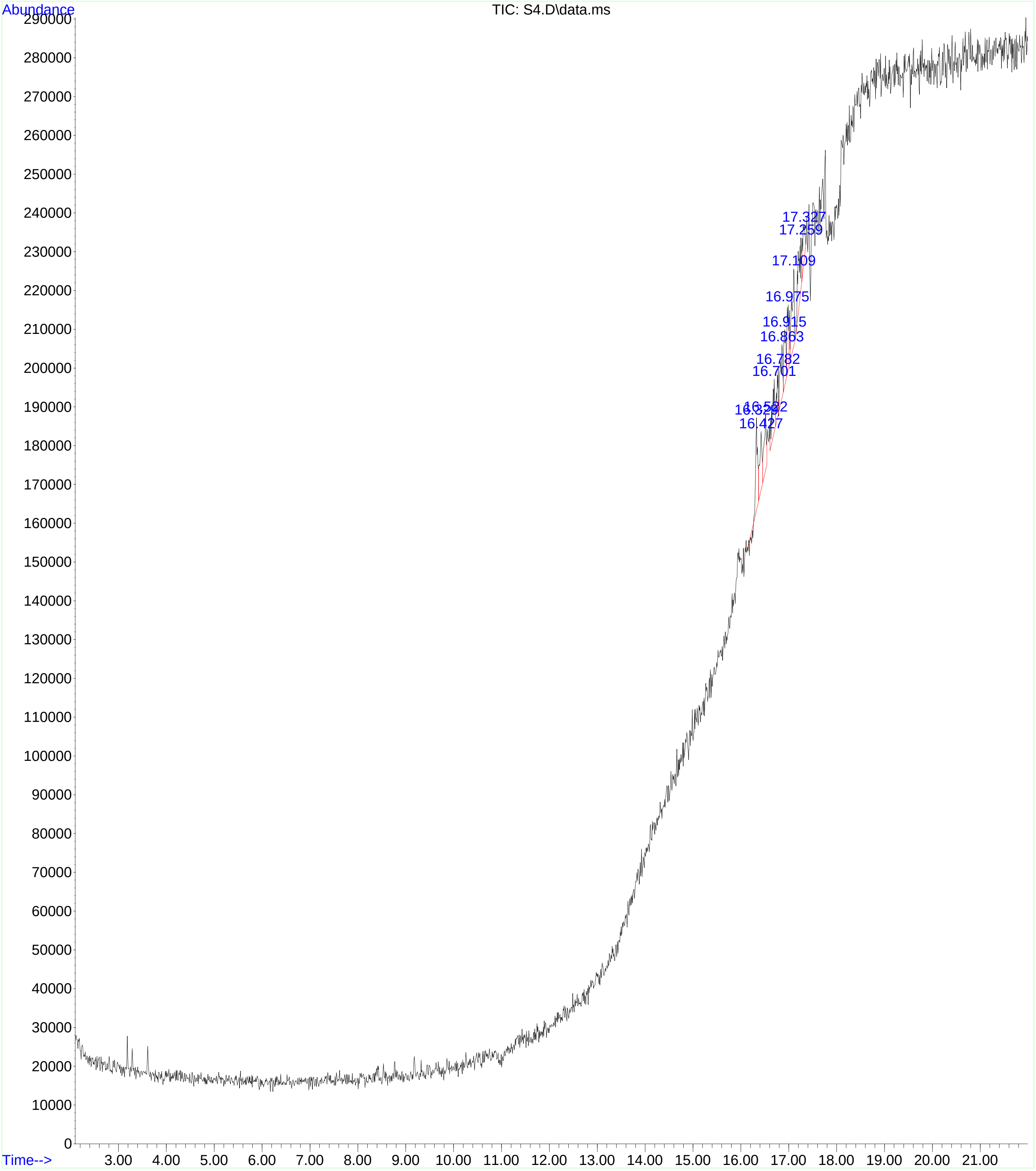

Library Search Report - ChemStation Integrator

Unknown Spectrum based on Apex

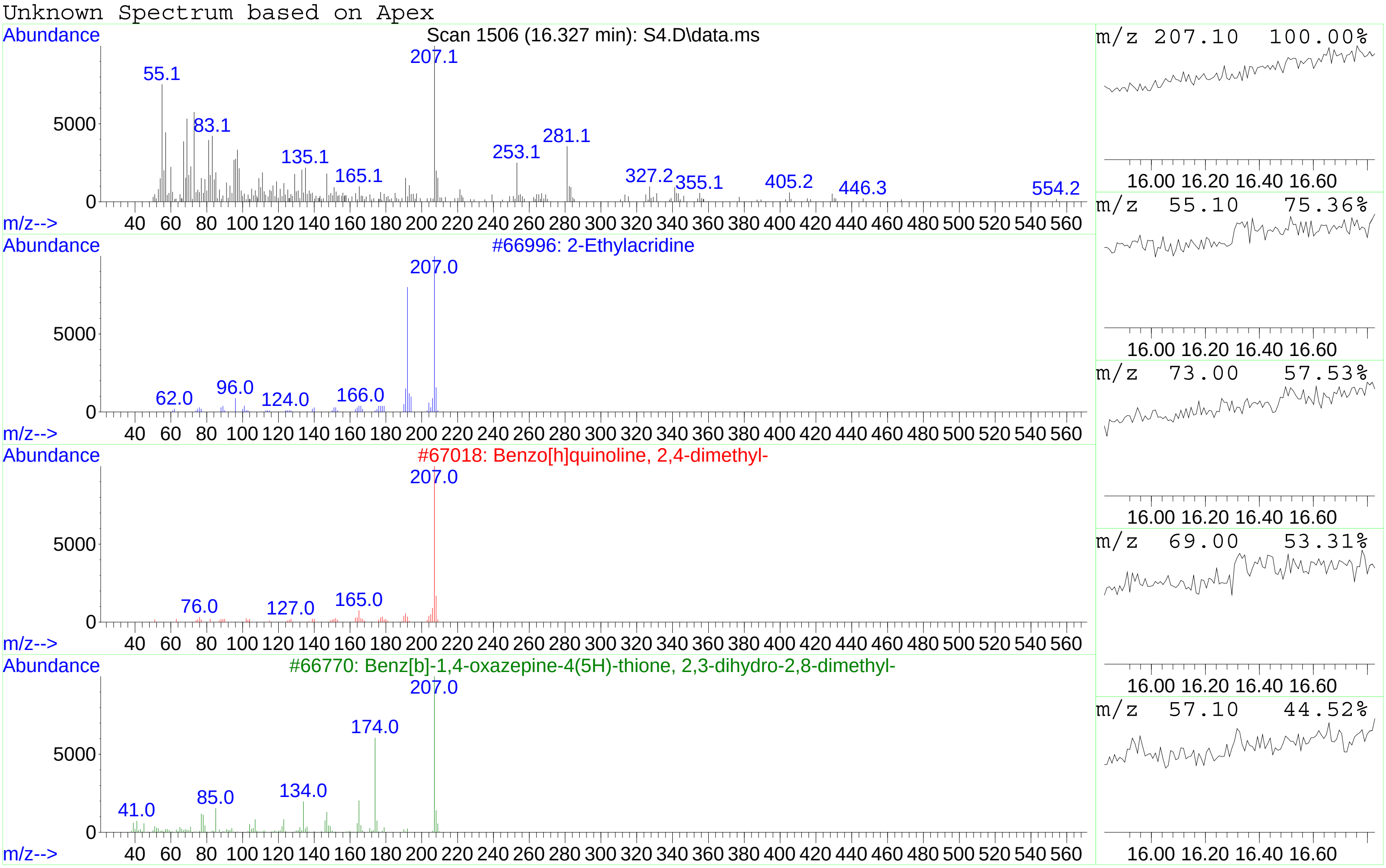

Data File: D:\Data\anjac\2015\ANJAC\noorul islam univ\S4.D
Sample : S4

Peak Number: 1 at 16.327 min Area: 520353 Area % 10.11

The 3 best hits from each library.	Ref\#	CAS\#	Qual
D:\MassHunter\Library\NIST11.L			
1 2-Ethylacridine	66996	055751-83-2	50
2 Benzo[h]quinoline, 2,4-dimethyl-	67018	000605-67-4	46
3 Benz[b]-1,4-oxazepine-4(5H)-thio...	66770	1000258-63-4	45

Unknown Spectrum based on Apex

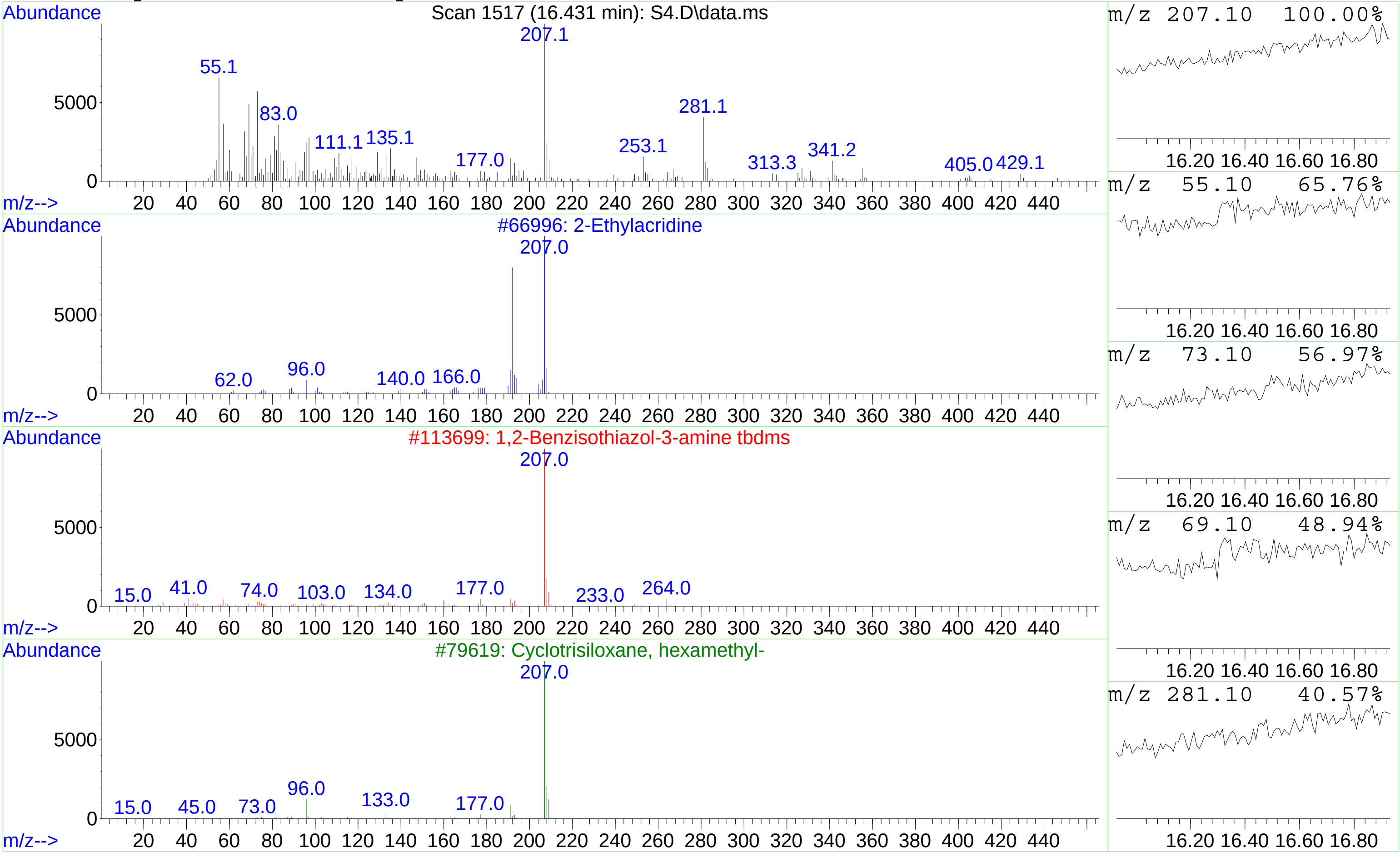

Data File: D:\Data\anjac\2015\ANJAC\noorul islam univ\S4.D
Sample : S4

Peak Number: 2 at 16.431 min Area: 490749 Area % 9.54

The 3 best hits from each library.	Ref\#	CAS\#	Qual
D:\MassHunter\Library\NIST11.L			
1 2-Ethylacridine	66996	055751-83-2	38
2 1,2-Benzisothiazol-3-amine tbdms	113699	1000332-57-2	38
3 Cyclotrisiloxane, hexamethyl-	79619	000541-05-9	35

Library Search Report - ChemStation Integrator

Unknown Spectrum based on Apex

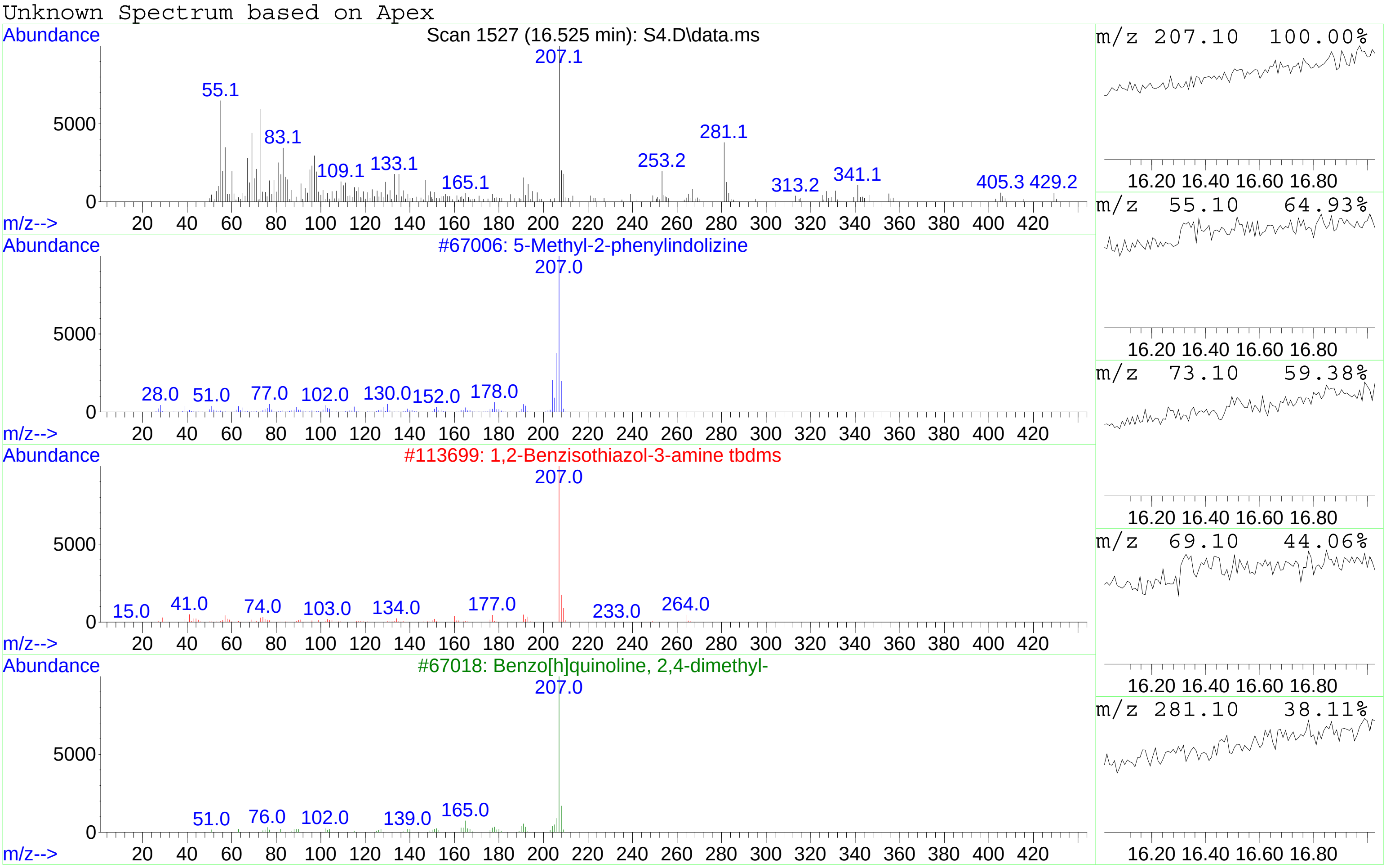

Data File: D:\Data\anjac\2015\ANJAC\noorul islam univ\S4.D
Sample : S4

Peak Number: 3 at 16.525 min Area: 458790 Area % 8.92

The 3 best hits from each library.	Ref\#	CAS\#	Qual
D:\MassHunter\Library\NIST11.L			
1 5-Methyl-2-phenylindolizine	67006	036944-99-7	38
2 1,2-Benzisothiazol-3-amine tbdms	113699	1000332-57-2	38
3 Benzo[h]quinoline, 2,4-dimethyl-	67018	000605-67-4	38

Unknown Spectrum based on Apex

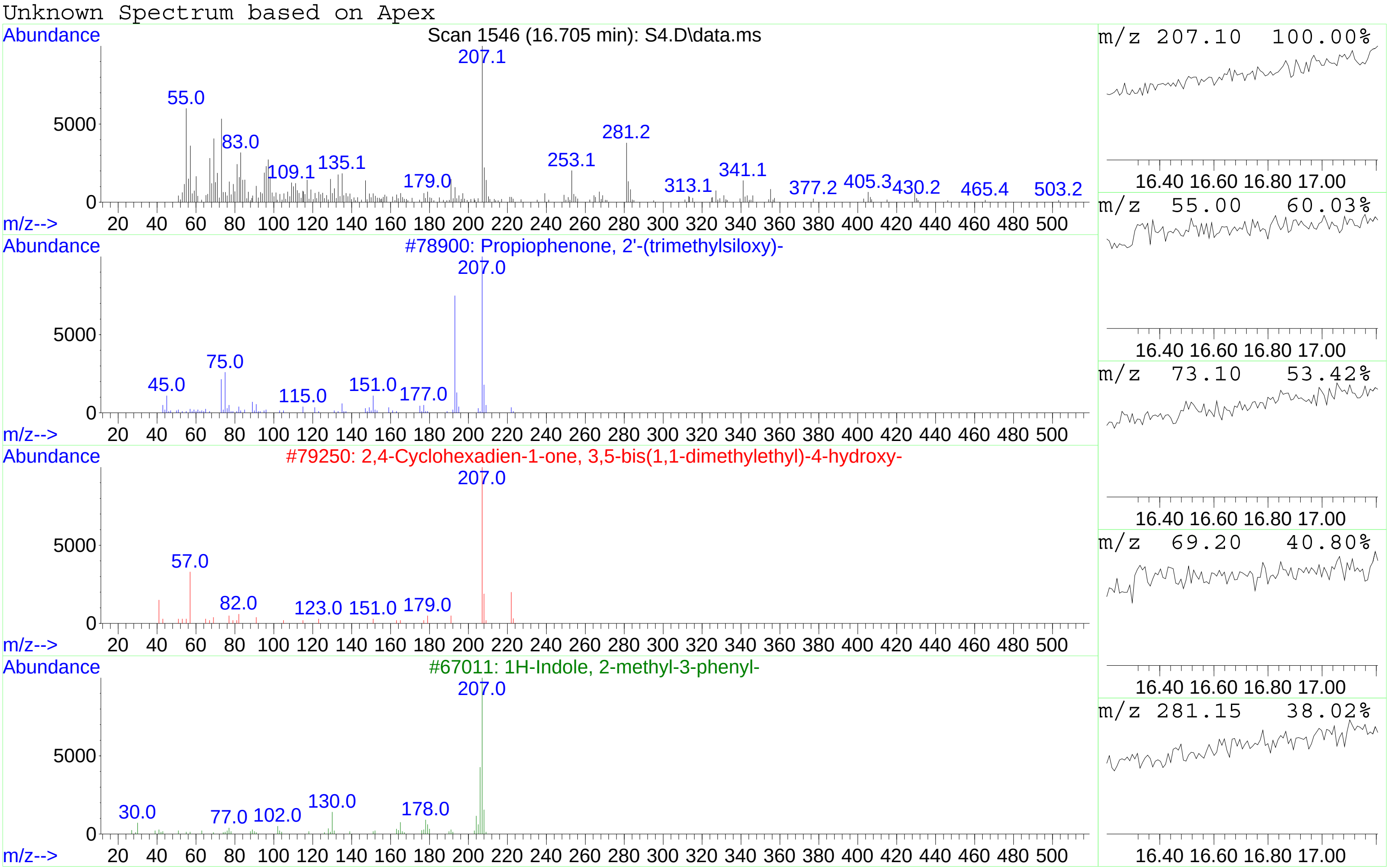

Data File: D:\Data\anjac\2015\ANJAC\noorul islam univ\S4.D
Sample : S4

Peak Number: 4 at 16.705 min Area: 487392 Area % 9.47

The 3 best hits from each library.	Ref#	CAS#	Qual
D:\MassHunter\Library\NIST11.L			
1 Propiophenone, 2'-(trimethylsilo...	78900	033342-87-9	45
2 2,4-Cyclohexadien-1-one, 3,5-bis...	79250	054965-43-4	45
3 1H-Indole, 2-methyl-3-phenyl-	67011	004757-69-1	45

Library Search Report - ChemStation Integrator

Unknown Spectrum based on Apex

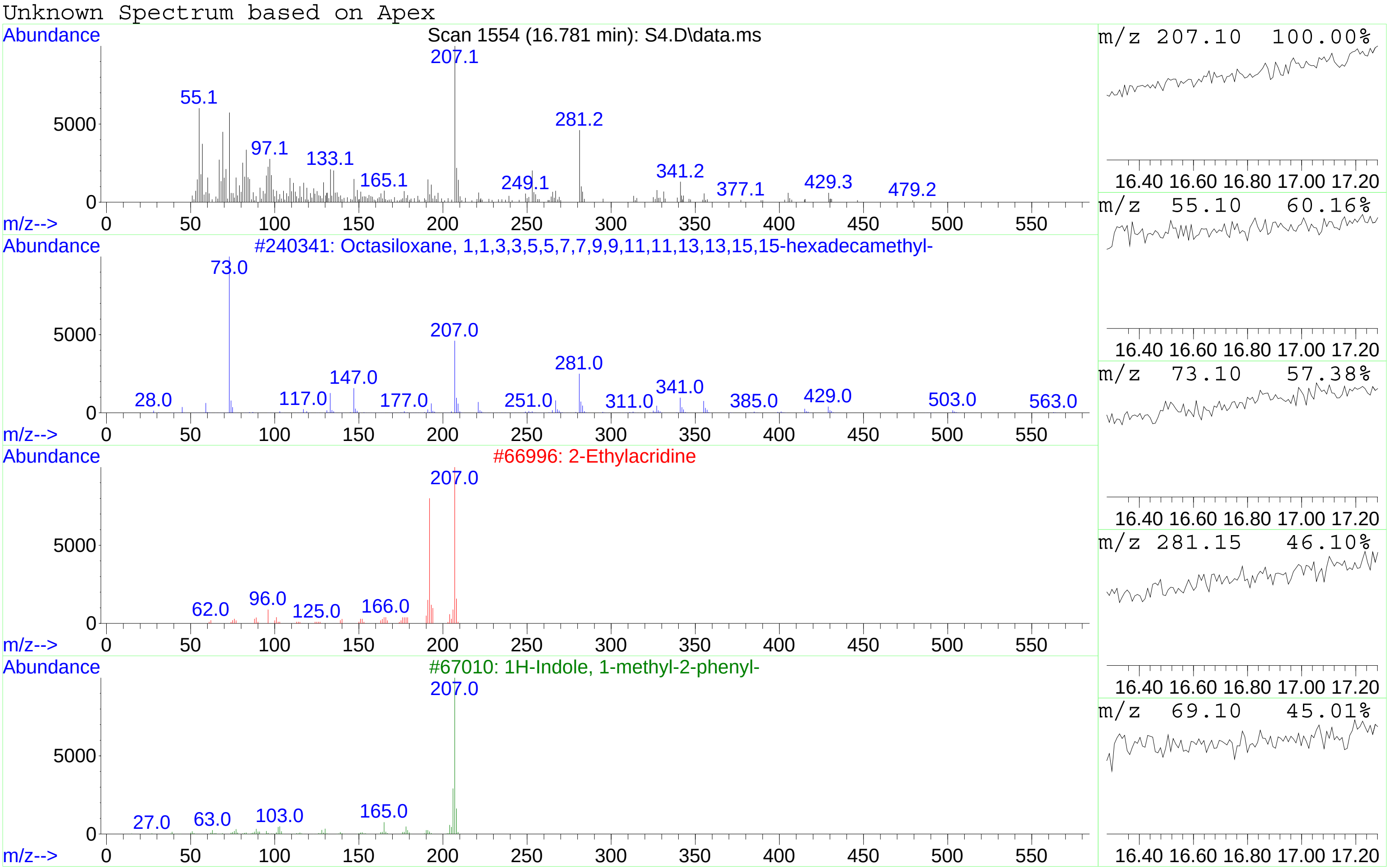

Data File: D:\Data\anjac\2015\ANJAC\noorul islam univ\S4.D
Sample : S4

Peak Number: 5 at 16.781 min Area: 292451 Area % 5.68

The 3 best hits from each library.	Ref#	CAS#	Qual
D:\MassHunter\Library\NIST11.L			
1 Octasiloxane, 1,1,3,3,5,5,7,7,9,...	240341	019095-24-0	53
2 2-Ethylacridine	66996	055751-83-2	43
3 1H-Indole, 1-methyl-2-phenyl-	67010	003558-24-5	43

Unknown Spectrum based on Apex

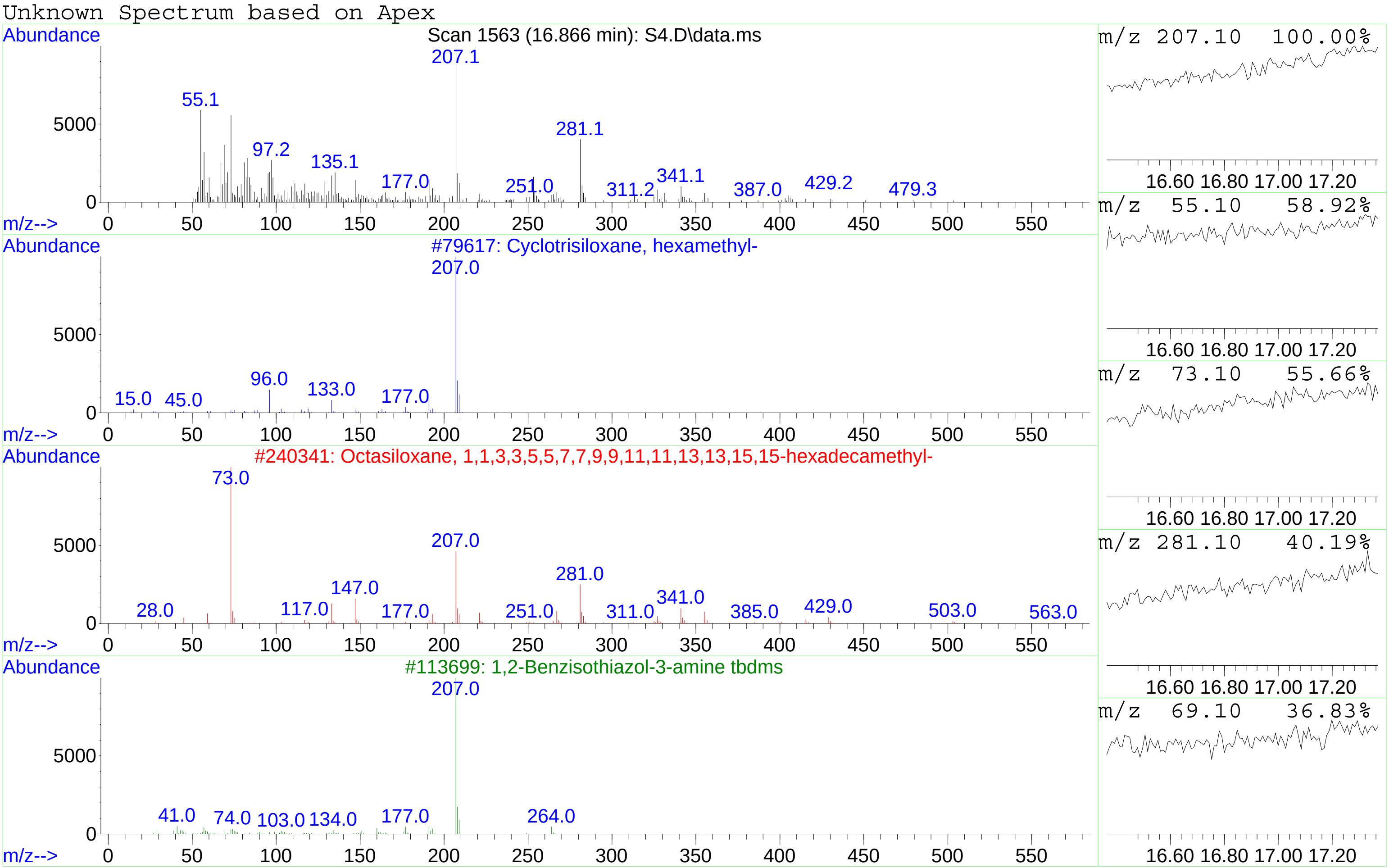

Data File: D:\Data\anjac\2015\ANJAC\noorul islam univ\S4.D
Sample : S4

Peak Number: 6 at 16.866 min Area: 464258 Area % 9.02

The 3 best hits from each library.	Ref\#	CAS\#	Qual
D:\MassHunter\Library\NIST11.L			
1 Cyclotrisiloxane, hexamethyl-	79617	000541-05-9	43
2 Octasiloxane, 1,1,3,3,5,5,7,7,9,...	240341	019095-24-0	43
3 1,2-Benzisothiazol-3-amine tbdms	113699	1000332-57-2	41

Library Search Report - ChemStation Integrator

Unknown Spectrum based on Apex

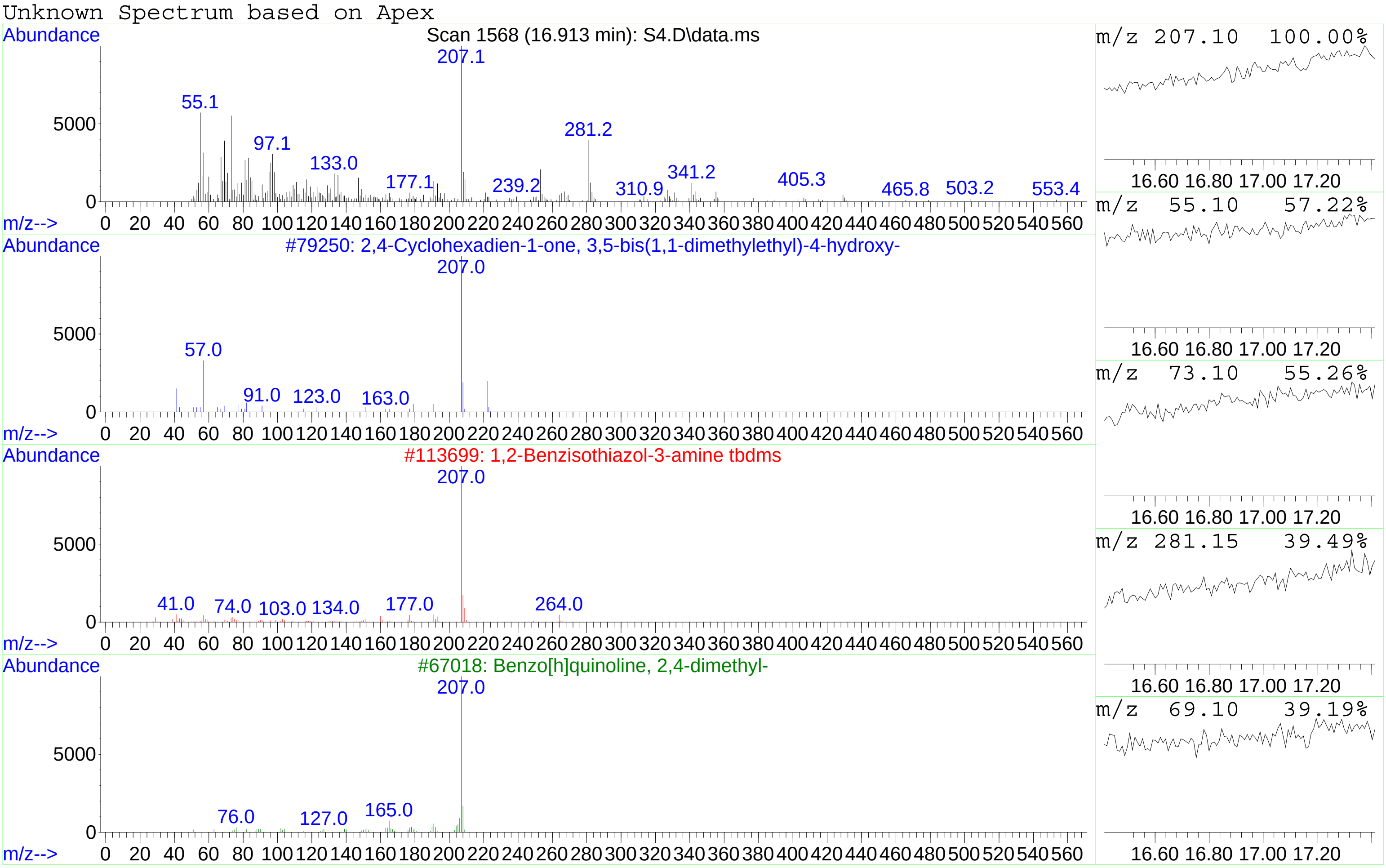

Data File: D:\Data\anjac\2015\ANJAC\noorul islam univ\S4.D
Sample : S4

Peak Number: 7 at 16.913 min Area: 413091 Area % 8.03

The 3 best hits from each library.	Ref\#	CAS\#	Qual
D:\MassHunter\Library\NIST11.L			
1 2,4-Cyclohexadien-1-one, 3,5-bis...	79250	054965-43-4	50
2 1,2-Benzisothiazol-3-amine tbdms	113699	1000332-57-2	45
3 Benzo[h]quinoline, 2,4-dimethyl-	67018	000605-67-4	45

Unknown Spectrum based on Apex

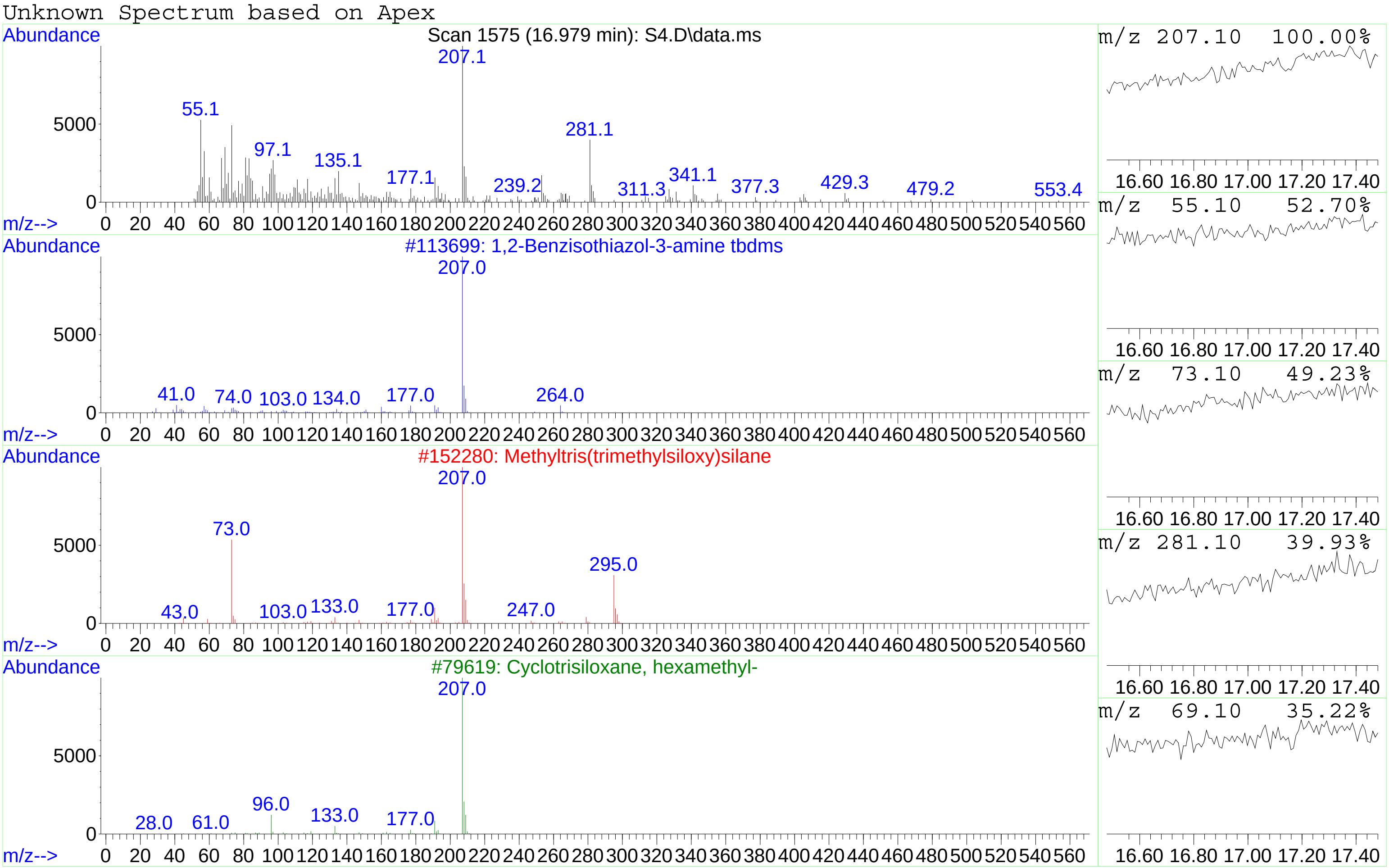

Data File: D:\Data\anjac\2015\ANJAC\noorul islam univ\S4.D
Sample : S4

Peak Number: 8 at 16.979 min Area: 375781 Area % 7.30

The 3 best hits from each library.	Ref#	CAS#	Qual
D:\MassHunter\Library\NIST11.L			
1 1,2-Benzisothiazol-3-amine tbdms	113699	1000332-57-2	43
2 Methyltris(trimethylsiloxy)silane	152280	017928-28-8	38
3 Cyclotrisiloxane, hexamethyl-	79619	000541-05-9	38

Unknown Spectrum based on Apex

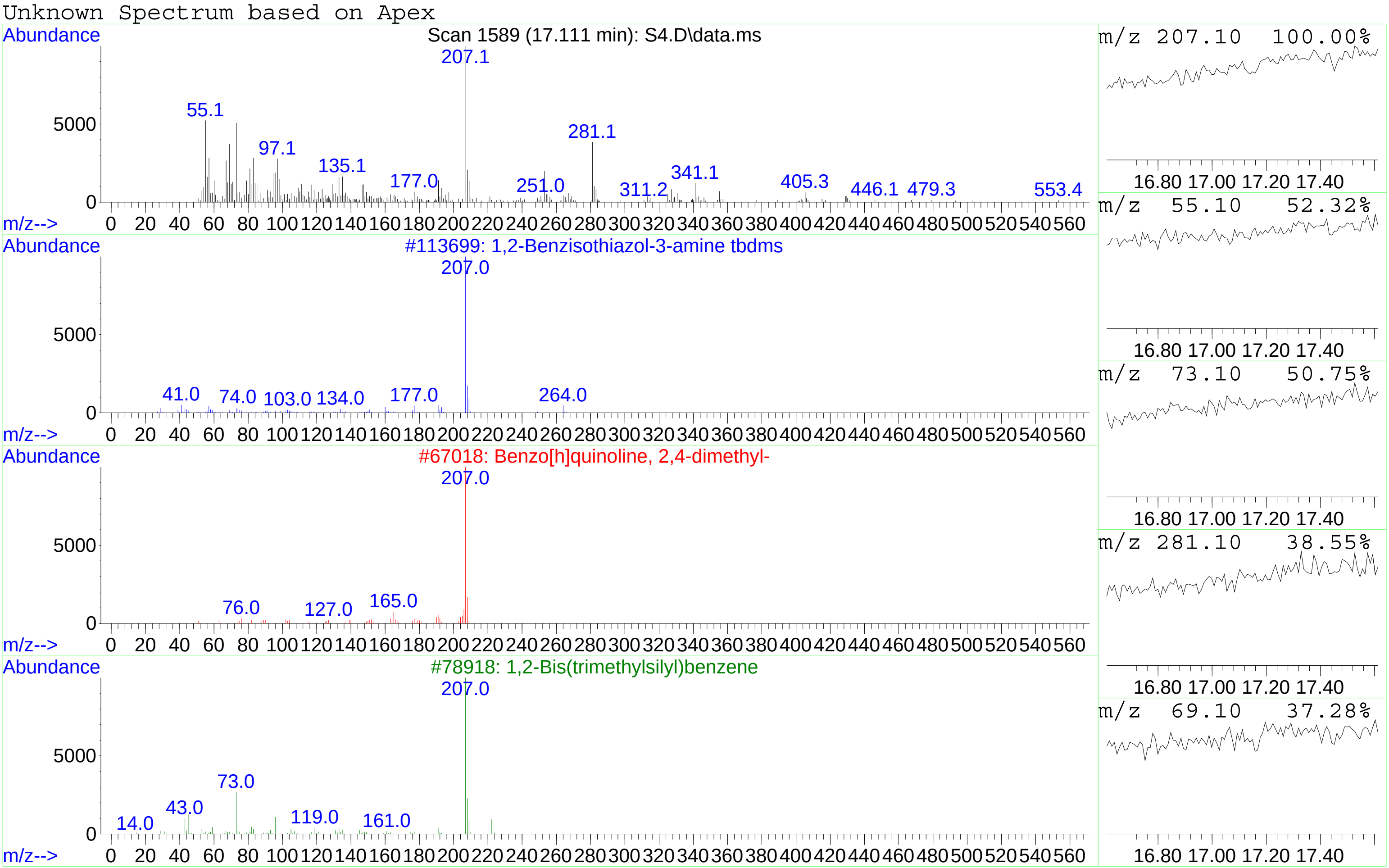

Data File: D:\Data\anjac\2015\ANJAC\noorul islam univ\S4.D
Sample : S4

Peak Number: 9 at 17.111 min Area: 677981 Area % 13.17

The 3 best hits from each library.	Ref\#	CAS\#	Qual
D:\MassHunter\Library\NIST11.L			
1 1,2-Benzisothiazol-3-amine tbdms	113699	1000332-57-2	45
2 Benzo[h]quinoline, 2,4-dimethyl-	67018	000605-67-4	43
3 1,2-Bis(trimethylsilyl)benzene	78918	017151-09-6	43

Library Search Report - ChemStation Integrator

Unknown Spectrum based on Apex

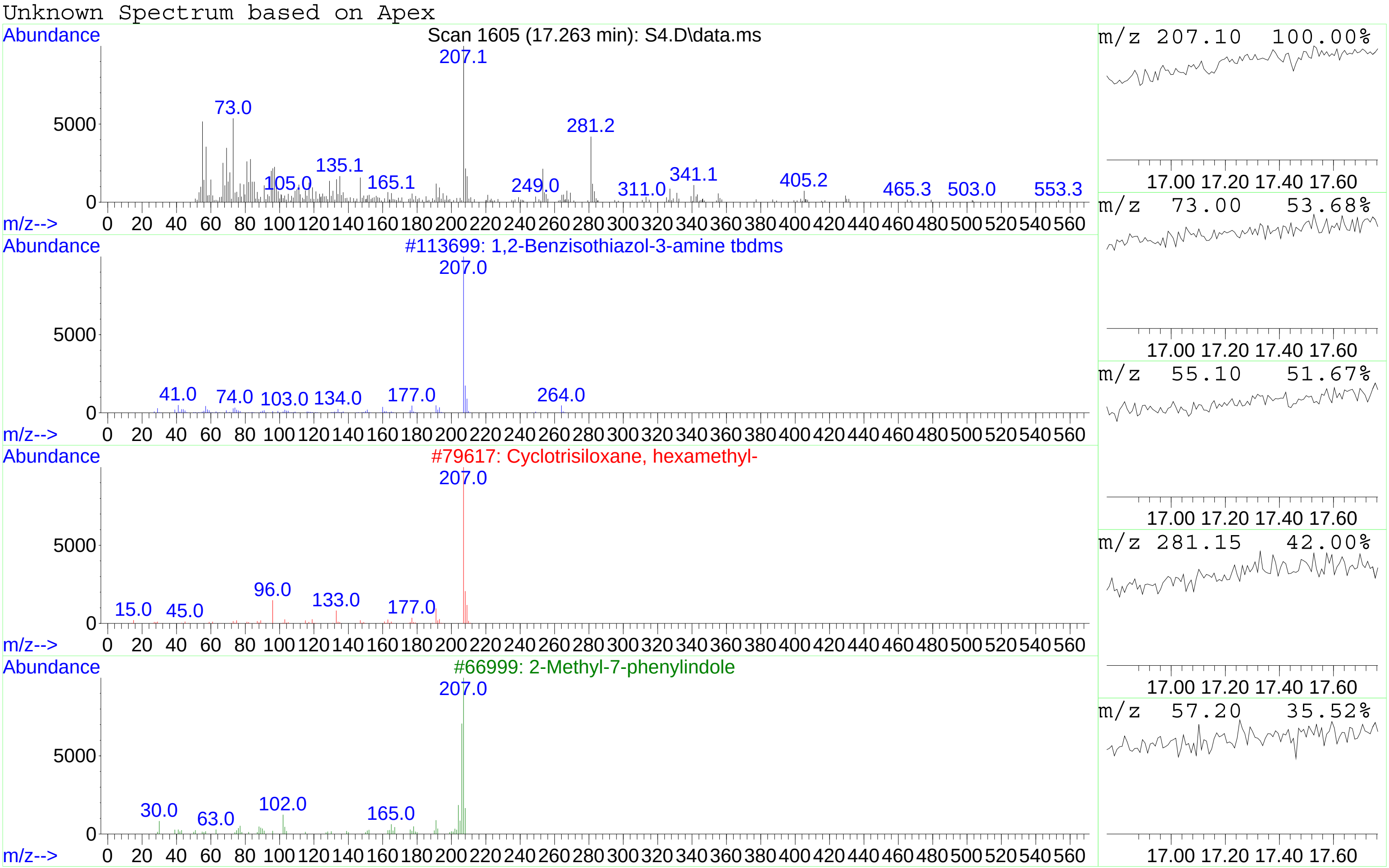

Data File: D:\Data\anjac\2015\ANJAC\noorul islam univ\S4.D
Sample : S4

Peak Number: 10 at 17.263 min Area: 604813 Area % 11.75

The 3 best hits from each library.	Ref\#	CAS\#	Qual
D:\MassHunter\Library\NIST11.L			
1 1,2-Benzisothiazol-3-amine tbdms	113699	1000332-57-2	49
2 Cyclotrisiloxane, hexamethyl-	79617	000541-05-9	46
3 2-Methyl-7-phenylindole	66999	001140-08-5	43

Library Search Report - ChemStation Integrator

Unknown Spectrum based on Apex

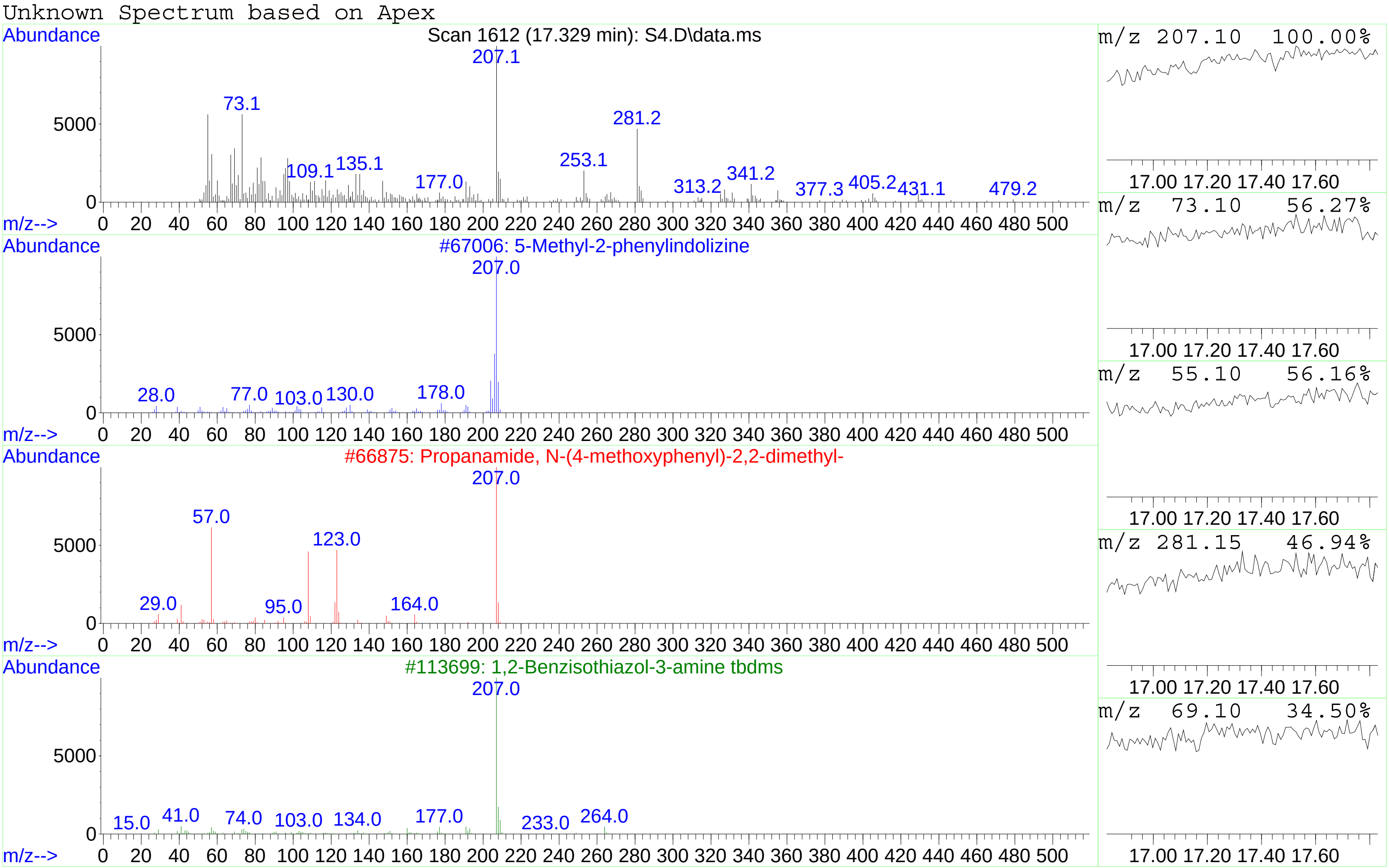

Data File: D:\Data\anjac\2015\ANJAC\noorul islam univ\S4.D
Sample : S4

Peak Number: 11 at 17.329 min Area: 360471 Area % 7.00

The 3 best hits from each library.	Ref\#	CAS\#	Qual
D:\MassHunter\Library\NIST11.L			
1 5-Methyl-2-phenylindolizine	67006	036944-99-7	38
2 Propanamide, N-(4-methoxyphenyl)...	66875	056619-94-4	38
3 1,2-Benzisothiazol-3-amine tbdms	113699	1000332-57-2	38
